# Asthma and Farm Exposures in a Cohort of Rural Iowa Children

**DOI:** 10.1289/ehp.7240

**Published:** 2004-12-07

**Authors:** James A. Merchant, Allison L. Naleway, Erik R. Svendsen, Kevin M. Kelly, Leon F. Burmeister, Ann M. Stromquist, Craig D. Taylor, Peter S. Thorne, Stephen J. Reynolds, Wayne T. Sanderson, Elizabeth A. Chrischilles

**Affiliations:** ^1^Department of Occupational and Environmental Health, University of Iowa College of Public Health, Iowa City, Iowa, USA; ^2^Center for Health Research, Kaiser Permanente Northwest, Portland, Oregon, USA; ^3^National Health and Environmental Effects Research Laboratory, Human Studies Division, Epidemiology and Biomarkers Branch, U.S. Environmental Protection Agency, Research Triangle Park, North Carolina, USA; ^4^Department of Biostatistics, University of Iowa College of Public Health, Iowa City, Iowa, USA; ^5^Department of Environmental and Radiological Health Sciences, Colorado State University College of Veterinary Medicine and Biomedical Sciences, Fort Collins, Colorado, USA; ^6^Department of Epidemiology, University of Iowa College of Public Health, Iowa City, Iowa, USA

**Keywords:** agricultural occupational exposures, ammonia, animal feeding operations, asthma, asthma diagnosis and treatment, asthma health care policy, asthma school screening, asthma underdiagnosis, asthma undertreatment, children, chronic wheeze, cough with exercise, farming, genetic selection, hydrogen sulfide, hygiene hypothesis, odor, rural

## Abstract

Epidemiologic studies of farm children are of international interest because farm children are less often atopic, have less allergic disease, and often have less asthma than do nonfarm children—findings consistent with the hygiene hypothesis. We studied a cohort of rural Iowa children to determine the association between farm and other environmental risk factors with four asthma outcomes: doctor-diagnosed asthma, doctor-diagnosed asthma/medication for wheeze, current wheeze, and cough with exercise. Doctor-diagnosed asthma prevalence was 12%, but at least one of these four health outcomes was found in more than a third of the cohort. Multivariable models of the four health outcomes found independent associations between male sex (three asthma outcomes), age (three asthma outcomes), a personal history of allergies (four asthma outcomes), family history of allergic disease (two asthma outcomes), premature birth (one asthma outcome), early respiratory infection (three asthma outcomes), high-risk birth (two asthma outcomes), and farm exposure to raising swine and adding antibiotics to feed (two asthma outcomes). The high prevalence of rural childhood asthma and asthma symptoms underscores the need for asthma screening programs and improved asthma diagnosis and treatment. The high prevalence of asthma health outcomes among farm children living on farms that raise swine (44.1%, *p* = 0.01) and raise swine and add antibiotics to feed (55.8%, *p* = 0.013), despite lower rates of atopy and personal histories of allergy, suggests the need for awareness and prevention measures and more population-based studies to further assess environmental and genetic determinants of asthma among farm children.

Most epidemiologic studies of childhood asthma have been conducted among inner-city or urban populations and have found asthma prevalence to vary by location, likely attributable to differing environmental exposures [International Study of Asthma and Allergies in Children (ISAAC) Steering Committee 1998]. Studies of rural childhood asthma are of particular interest because they have consistently reported that farm children are less often atopic (Braun-Fahrlander et al. 1999; Downs et al. 2001; Riedler et al. 2000, 2001), have lower rates of allergic diseases (Braun-Fahrlander et al. 1999; Kilpelainen et al. 2000; Riedler et al. 2000, 2001; Von Ehrenstein et al. 2000; Wickens et al. 2002), and in several reports also have lower rates of asthma (Ernst and Cormier 2000; Kilpelainen et al. 2000; Riedler et al. 2000, 2001; Von Ehrenstein et al. 2000). These findings are consistent with the hygiene hypothesis, which posits that childhood allergy risk is immunologically modulated in early life by exposure to infectious agents. However, several studies have not found positive associations between asthma and asthma symptoms among children and farm exposures, raising questions regarding the influence of unmeasured risk factors and/or selection in these cross-sectional studies (Chrischilles et al. 2004; Downs et al. 2001; Salam et al. 2004; Wickens et al. 2002).

It is recognized that asthma risk is conveyed by a complex interaction of genetic and environmental determinants, which makes the epidemiologic investigation of farm-related asthma difficult (Douwes et al. 2001; Niven 2003; Schwartz 2001). International studies of childhood asthma among farm children have typically measured atopy to gauge genetic predisposition to asthma but have less consistently described and measured farm environment risk factors, often using endotoxin as an indicator of exposure to infectious agents early in life. Although endotoxin is a ubiquitous exposure in agriculture, its concentration varies within and between farm types, and it is but one of many agricultural respiratory exposures children may encounter (Douwes et al. 2003; Reynolds et al. 1996; Schenker et al. 1998).

Over the last three decades, the development of a vertically integrated livestock industry has significantly reduced the number of U.S. family farms raising hogs, poultry, and cattle but has rapidly increased the number of large animal-feeding operations (AFOs) (National Academy of Sciences 2003). Although inflammatory airway diseases, including asthma, chronic bronchitis, organic dust toxic syndrome, and progressive airway obstruction, are now well documented among AFO workers (Schenker et al. 1998), there has been much less research regarding exposures and health outcomes among AFO-exposed children and community-based residents (Reynolds et al. 1997a; Salam et al. 2004; Thu et al. 1997; Wing and Wolf 2000).

The Keokuk County Rural Health Study (KCRHS) is a large, population-based study of a cohort of rural families living in an intensely agricultural region of southeastern Iowa (Merchant et al. 2002). The aim of the present study was to estimate asthma prevalence and assess whether farm exposures result in less atopy, less allergic disease, and less asthma, while taking into account multiple personal and other environmental risk factors, among this cohort of farm children.

## Materials and Methods

### The study population.

This study reports data on children from birth through 17 years of age collected in round 1 of the KCRHS, which began in 1994 and ended in 1998. Keokuk County was chosen because it is intensely agricultural and entirely rural. A stratified, random sample that identified households from farm, town, and rural nonfarm locations was used. A total of 2,496 eligible households were identified. Details regarding the sampling methodology and survey methods have been reported previously (Merchant et al. 2002). All members of enrolled households were invited to a centrally located research facility for interviews, and all adults and children ≥8 years of age were invited for medical examinations. One adult per household was interviewed by a trained interviewer about the health of all of the children (from birth but < 18 years of age) living in the household.

### Questionnaire.

The childhood respiratory questionnaire chosen for this study was that used in University of Southern California studies of childhood asthma in Los Angeles (Peters et al. 1999). We used four asthma outcomes to estimate asthma prevalence—doctor-diagnosed asthma, asthma/medication for wheeze (doctor-diagnosed asthma and/or medication for wheeze in the last 12 months), current wheeze, and cough with exercise. These four asthma outcomes, severe symptoms consistent with asthma, atopy, an early respiratory illness, and a high-risk birth are fully defined in the definition section of the online version this article. The parent’s response to the questionnaire also provided information regarding parental farm exposures, maternal smoking during pregnancy, household exposure to tobacco smoke, parental education, and household income.

### Clinical assessment.

Children ≥8 years of age were invited to complete a medical examination that included skin prick testing (SPT), spirometry, methacholine challenge testing, and height and weight measurements to calculate 95th percentile body mass index (kilograms per square meter) (Must et al. 1991). A total of 18 aeroallergens common to the Midwest, a histamine-positive and normal saline-negative control, were used for SPTs. Common rural aeroallergens included tree pollen mix, grass pollen mix, ragweed pollen, weed pollen mix, cockroach mix, mold mix, insect mix, caddis fly/moth/mayfly mix, cat pelt, dog hair, mouse and rat mix, and dust mite Der f and Der p mix. Farm aeroallergens included grain dust mix or grain smut mix, soybean dust or soybean whole grain, cattle hair, horse hair, chicken feathers, and turkey feathers. Children taking antihistamines and other medications known to affect skin test results, those with histories of past systemic reactions to allergy skin testing, and any participant who might have been pregnant were excluded from skin testing. A wheal ≥3 mm in diameter was defined as a positive reaction; subjects were considered atopic by SPT if they had a positive reaction to any two of the allergens tested. Spirometry was completed on a rolling-seal spirometer that conformed to American Thoracic Society (1995) guidelines. Contraindications to methacholine testing included participants with a baseline forced expiratory volume in 1 sec (FEV_1_) of < 70% of predicted or FEV_1_ < 1.5 L, pregnancy or suspected pregnancy, lactation, current use of a β-adrenergic blocking agent, and a decline in FEV_1_ of ≥15% to the diluent. Methacholine was administered by dosimeter in five serial doses of 0.025, 0.25, 2.5, 10.0, and 25.0 mg/mL, with 3 min between doses (Crapo et al. 2000). Bronchial hyperresponsiveness was defined as having a drop in FEV_1_ of ≥20% from the postsaline control (PC20), following inhalation of ≤8 mg/mL of methacholine (Anto 1998; Crapo et al. 2000).

### Serum analysis.

Sera were collected from subjects (*n* = 217) at the time of SPT and analyzed for total and specific IgE. Total IgE was measured by immunoassay using murine monoclonal anti-human IgE as the capture antibody (CLB, Sanguin Blood Supply Foundation, Amsterdam, the Netherlands), rabbit anti-human IgE as the second antibody (Dako, Corp., Carpinteria, CA), and peroxidase-conjugated donkey anti-rabbit IgG as the labeling antibody (Research Diagnostics, Inc., Flanders, NJ) in a TMB substrate system (Pierce Endogen, Rockford, IL). Standard curves were generated using an IgE CAP system standards (Pharmacia Diagnostics, Uppsala, Sweden) with the standard curve from 0.02 to 10 kU/L. Sera were studied at initial dilutions of 1:20, 1:40, 1:80, and 1:160, with higher dilutions run for high IgE sera. Individuals were considered to be atopic by IgE if their total IgE was ≥60 kU/L (Contreras et al. 2003).

### Environmental assessment.

An industrial hygienist visited each household shortly after the clinic visit and completed a home environmental questionnaire and checklist, when applicable a farm environmental questionnaire and farm environmental checklist, and measurement of a limited number of environmental parameters. Details of these environmental assessments have been published previously (Park et al. 2003; Reynolds et al. 1997b). Assessments of specific environmental exposures were taken from these instruments, including several farm operation questions, livestock and antibiotics in animal feed questions, and questions regarding gas stoves, heating with wood, exposure to pesticides, exposure to cats and dogs as pets, and dehumidifier use.

Household type was determined at the time of the child’s birth from the biologic mother’s reproductive history questionnaire and through follow-up phone interviews with the biologic mother regarding residence type (farm, rural nonfarm, or home) at the time of birth. Children’s various farm tasks and the age each task was first performed were determined from a questionnaire on childhood tasks from available KCRHS round 2 data and from follow-up phone administration of this questionnaire to round 1 participants who had not participated in round 2.

### Statistical analysis.

Chi-squared tests and analysis of variance were used to evaluate any differences among demographic, personal, and environmental risk factors for farm, rural non-farm, and town households. Univariable logistic regression was used to identify variables that were significant (*p* < 0.1) for doctor-diagnosed asthma, asthma/medication for wheeze, chronic wheeze, and cough with exercise. Multivariable logistic regression was then used to identify significant (*p* < 0.05) variables in the final models.

Initial data analyses was performed with SAS (version 8; SAS Institute, Inc., Cary, NC) software. SUDAAN software (Research Triangle Institute, Research Triangle Park, NC) was then used to adjust variance estimates for potential intrahousehold correlation resulting from the inclusion of more than one child per household.

The study was approved annually by The University of Iowa institutional review board. A parent or legally authorized representative of each child participant provided written informed consent. Children 8–17 years of age gave their assent.

## Results

### Cohort description.

Of the 2,496 Keokuk County households eligible for this study, 1,675 households (67.1%) initially contacted by letter and telephone agreed to participate immediately or to be contacted at a later date. Enrollment stopped when the goal of 1,000 households was reached. A total of 1,004 households (336 farm, 206 rural non-farm, 462 town households) enrolled and participated in round 1 of the study.

The cohort, which consisted of 644 children (224 farm, 155 rural nonfarm, and 265 town), did not differ in age among household types, was somewhat overrepresented by boys in farm and rural nonfarm households, and was 97.7% Caucasian. Of the 336 farms in the cohort, 109 had children. Complete data on all farming characteristics were available on 89 farms with children and on 172 farms without children. These farms produced primarily corn, soybeans, and hogs but very few other livestock. Farms with children were somewhat smaller (434 total acres in production) than farms without children (468 total acres in production) but were otherwise similar, except that farms with children on average raised more hogs (298 vs. 141, *p* = 0.03). Fifty percent of farm children were reported by a parent to perform tasks around hogs, compared with ≤16% for rural non-farm or town children, whereas 40% of farm children were reported to perform tasks around cows compared with ≤13% for rural nonfarm or town children.

### Health outcomes.

Ninety-five percent of the children’s data were provided by the child’s biologic mother or female guardian. Complete data on asthma outcomes were available on 610 children. Concordance between the four asthma outcomes varied from strong to weak: doctor-diagnosed asthma (asthma/medication for wheeze κ = 0.81, *p* < 0.0001; current wheeze κ = 0.31, *p* < 0.0001; cough with exercise κ = 0.26, *p* < 0.0001), asthma/medication for wheeze (current wheeze κ = 0.53, *p* < 0.0001; cough with exercise κ = 0.39, *p* = 0.11; current wheeze and cough with exercise κ = 0.27, *p* = 0.73). Only 4.4% of participants were captured by all four asthma outcomes, whereas 33.6% of all 610 participants were captured by at least one asthma outcome. Children with doctor-diagnosed asthma included only a third (8 of 24) of the children with severe symptoms consistent with asthma, whereas children with any one of the four asthma outcomes captured 23 of 24 children with severe symptoms. Of the 394 children 8–17 years of age, 351 (89.1%) had SPT, 347 (88.1%) had pulmonary function tests, and 215 (61.2%) agreed to have blood drawn for sera. Agreement between total individual IgE and SPT results (*Aspergillus*, cat hair, cockroach, weed mix, tree pollen, Der p, and Der f) ranged from 72.8 to 89.1%.

Children who were born on a farm had a lower prevalence of atopy (IgE), a lower prevalence of diagnosed allergies and a higher forced vital capacity (likely attributable to hyperinflation) (Table 1). Children who currently lived on a farm were somewhat more likely to be boys and somewhat less likely to have diagnosed allergies (Table 1).

A very high proportion of children who lived on a farm at the time of study (currently lives on a farm) were born when their parents lived on a farm (born on a farm) and continued to live on a farm (data for those who lived on a farm during the first year of life or through age 5, or had a parent who continued to work on a farm, were also analyzed but not reported). Because univariable associations were similar for all farm versus nonfarm groups, only comparisons of born on a farm and currently living on a farm exposure results are presented (Table 2). Farm children were consistently exposed to less tobacco smoke but were more often exposed to wood stoves, conditions resulting in dehumidifier use, cats as pets, and application of pesticides outside the home. Farm children’s parents were more often better educated and had a household annual income of ≥$20,000 (Table 2).

Univariable associations among the four asthma outcomes and environmental risk factors are presented in Tables 3 and 4. A weak association was observed between doctor-diagnosed asthma and less parental education. A near significant association was observed between doctor-diagnosed asthma/medication for wheeze and living on a farm raising swine and a significant association with living on a farm that adds antibiotics to feed. No significant association was observed with environmental exposures and current wheeze, but significant negative associations were observed between cough with exercise and exposure to wood smoke and applied pesticides outside home in the last year, significant positive associations were observed with dogs as household pets, and near significant positive associations were observed with living on a swine farm and living on a farm that added antibiotics to feed. Tables 5 and 6 present univariable associations among the four asthma health outcomes and personal and clinical risk factors and health measures, which reveal similar association patterns but a few significant differences.

**Figure 1:**
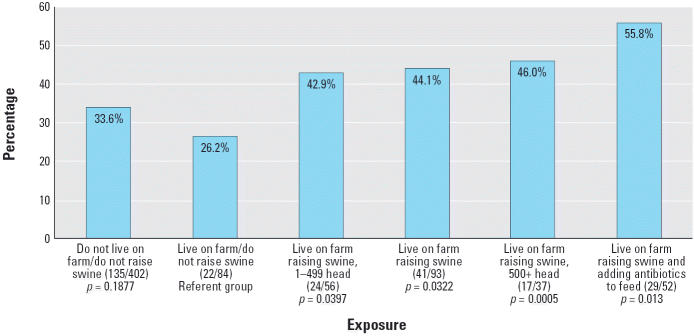
Prevalence of one or more asthma outcomes in rural Iowa children.

Multivariable models that included personal or environmental risk factors with univariable significance of *p* < 0.1 for any of the four asthma outcomes are presented in Table 7. In addition to sex, age, history of allergies, family history of allergies, premature birth, early respiratory infection, and high-risk birth, an interaction term (living on a farm that raised swine and added antibiotics to feed) was independently associated with asthma/medication for wheeze, current wheeze (*p* = 0.06), and cough with exercise. Of farms that raised swine, 24 of 43 (55.8%) added antibiotics to feed. Of livestock farms that add antibiotics to feed, 24 of 31 farms or 77.4% raise swine. Those farms that add antibiotics to feed were found to have larger mean numbers of livestock than those that did not add antibiotics to feed (750 vs. 392 animals; *p* = 0.0002). Examination of children who lived on farms raising swine and adding antibiotics to feed found that 55.8% (*p* = 0.013) reported at least one of the four asthma outcomes (Figure 1).

## Discussion

This study reports uniformly high-prevalence estimates of asthma and asthma-related symptoms that are consistent with asthma prevalence observed in studies of U.S. urban populations (Bauer et al. 1999; ISAAC Steering Committee 1998). These high asthma prevalence estimates, and our finding of a high proportion (two-thirds) of children with severe symptoms consistent with asthma but without a doctor diagnosis of asthma, are consistent with the findings of our Rural Childhood Asthma Study (Chrischilles et al. 2004) and underscore the need for asthma screening programs, for improved rural health care provider asthma diagnostic and management skills, and for health policies that would improve access and insurance coverage for rural children.

A history of diagnosed allergies was found to be less common among children who lived on a farm in the first year of life, a finding consistent with many other studies of farm children (Braun-Fahrlander et al. 1999; Kilpelainen et al. 2000; Riedler et al. 2000, 2001; Von Ehrenstein et al. 2000). The three estimates of atopy also tended to be lower among children who lived on a farm in the first year of life, as reported by others (Braun-Fahrlander et al. 1999; Riedler et al. 2000, 2001). However, asthma and asthmalike symptom prevalences were found to be high and to not differ between children with farm exposures and those without farm exposures, unlike the findings of others (Ernst and Cormier 2000; Kilpelainen et al. 2000; Riedler et al. 2000, 2001; Von Ehrenstein et al. 2000), despite lower rates of allergic disease and atopy and a significantly lower exposure to household tobacco smoke among farm children. However, as depicted in Figure 1, these excesses are found only among children living on farms raising swine, whereas a lower prevalence of asthma was observed among farm children not raising swine compared with nonfarm children, which is consistent with the aforementioned studies.

Farms in Northern Europe tend to be smaller than Iowa farms and to have livestock that are often housed in immediate proximity to living quarters, and these farm families have been described as more traditional in their way of life. Farms in Canada, Australia, and New Zealand are described as larger but typically not as livestock intensive as Iowa farms (Downs et al. 2001; Ernst and Cormier 2000; Wickens et al. 2002). Keokuk County farm families do not live in immediate proximity to livestock buildings but do usually live on the same acreage, typically with many farm family members participating in the farm operation. It is common for young children to be exposed to farming operations, including AFOs, as they accompany a parent or sibling in assisting with farm tasks (Park et al. 2003). Farm children in Keokuk County were reported by their parents to be exposed as bystanders to farm tasks around livestock as early as 1 year of age; however, such tasks around livestock were typically done by male adolescents. Although no environmental measurements of farm task exposures were made, several studies conducted in Iowa document high levels of occupational exposures to respirable and total dust, endotoxin, hydrogen sulfide, and ammonia, which have been associated with asthma, chronic bronchitis, cross-shift declines in lung function, and progressive declines in lung function over time among those working in AFOs (Reynolds et al. 1996; Schenker et al. 1998; Schwartz et al. 1995). It is therefore probable that some swine-farm–exposed children had high exposures to endotoxin and other AFO exposures and that some of the asthma and asthma symptoms observed among these farm youth are attributable to occupational exposures.

Multivariable models for doctor-diagnosed asthma/medication for wheeze and cough with exercise found that raising swine and adding antibiotics to feed were independently associated with these health outcomes. Because farms that add antibiotics to feed were much larger than those that did not add antibiotics to feed, adding antibiotics to feed may serve as an indicator of larger swine operations. However, it is plausible that antibiotic exposures may be playing some causal role because antibiotics have been documented to be components of emissions from AFOs (Hamscher et al. 2003; Svendsen et al. 2003) and, when consumed for medical purposes, have been associated with childhood asthma (Wickens et al. 1999). These high asthma estimates make clear that on-farm exposure to swine production is associated with asthma among children living on these farms and that swine production contributes to the higher prevalence of asthma outcomes in this livestock-intensive rural community. More detailed assessment of the temporal relationships between childhood farm exposures, including measurements of endotoxin-laden dust, irritant gases, and antibiotics in relation to asthma estimates, is needed to further our understanding of these relationships.

Other events early in life, apart from farm exposures, including premature birth, a respiratory infection at ≤3 years of age, and high-risk birth, were independently associated with asthma outcomes in this study, also consistent with other studies of childhood asthma (Farooqi and Hopkin 1998; Von Mutius et al. 1993). These early-life risk factors, which did not differ between farm and nonfarm participants in this study, may confound assessment of farm exposures in populations where farm families are poorer and have less adequate prenatal health care.

Two studies of nonfarm infants have evaluated the role of endotoxin exposures early in life and have reported no relationship between endotoxin levels and atopy, allergic disease, and asthma (Bolte et al. 2003; Park et al. 2001), findings inconsistent with the hygiene hypothesis. Another contributing explanation, which has been recognized, but only indirectly assessed (Braun-Fahrlander et al. 1999; Downs et al. 2001; Ernst and Cormier 2000; Leynaert et al. 2001), is the potential unmeasured effect of systematic genetic selection of those susceptible to farm-related respiratory disease away from farming over successive generations. It is common for farm youth to leave the farm in Keokuk County, so much so that we have reported a significant deficit of asthma among adult farm men compared with other men in Keokuk County (Merchant et al. 2002).

Because indicators of asthma associated with common farm exposures are influenced by genotypic patterns (Arbour et al. 2000; Gilliland et al. 2004), epidemiologic studies of genotype among farm family generations could help define patterns of differential selection of atopic, allergic, and asthmatic members of farm families away from farming.

Limitations of this study include the relatively small numbers of children with clinical data. Also, this study was not designed to address the question of whether exposures to dust, irritant gases, and odors arising from AFOs may be associated with respiratory symptoms or health conditions among rural residents living in proximity to farms with AFOs. However, the few community-based studies of AFO exposures have reported higher rates of airway symptoms (Reynolds et al. 1997a; Thu et al. 1997; Wing and Wolf 2000), and significant peaks in asthma hospital visits have been observed following peak exposures to total reduced sulfur (for children) and to hydrogen sulfide (for adults) from a large animal waste treatment complex (Campagna et al. 2004). As the result of these findings and community complaints about odor, several states now regulate some combination of hydrogen sulfide, total reduced sulfur, ammonia, and odor. Given our finding of a high prevalence of asthma outcomes among farm children living on swine farms, it is clear that farm parents should be aware of this risk and take precautions to reduce childhood respiratory exposures from AFOs. Evaluation of asthma outcomes and environmental exposures among school children and rural residents living proximate to AFOs remains an important research priority.

## Figures and Tables

**Table 1:** Farm exposures for living on a farm [% (no./total) or mean ± SD], personal and family risk factors, and asthma outcomes.

Variable	Born on a farm	Not born on a farm	OR (95% CI)	*p*-Value	Currently lives on a farm	Does not currently live on a farm	OR (95% CI)	*p*-Value
Male sex	56.2 (122/217)	52.0 (196/377)	1.19 (0.84–1.67)	0.3277	58.5 (131/224)	51.0 (214/420)	1.36 (0.98–1.88)	0.0654
Age (years)	9.6 ± 5.0 (*n* = 217)	9.6 ± 4.9 (*n* = 377)	1.00 (0.96–1.04)	1.00	10.0 ± 4.9 (*n* = 224)	9.5 ± 4.9 (*n* = 420)	1.02 (0.98–1.06)	0.36
No. of siblings < 18 years of age	1.6 ± 1.2 (*n* = 217)	1.4 ± 1.0 (*n* = 377)	1.15 (0.87–1.53)	0.33	1.5 ± 1.2 (*n* = 224)	1.5 ± 1.0 (*n* = 420)	1.04 (0.77–1.40)	0.79
Atopy (IgE)	29.3 (24/82)	42.0 (50/119)	0.57 (0.31–1.04)	0.0661	32.5 (27/83)	38.8% (52/134)	0.76 (0.43–1.36)	0.3477
Atopy (SPT)	13.6 (15/110)	18.7 (34/182)	0.69 (0.34–1.40)	0.2926	18.6 (21/113)	17.5 (36/206)	1.08 (0.57–2.06)	0.8196
Atopy (by questionnaire)	21.2 (46/217)	22.8 (86/377)	0.91 (0.53–1.57)	0.7333	24.1 (54/224)	22.9 (96/420)	1.07 (0.61–1.90)	0.8122
Diagnosed allergies	10.8 (23/212)	17.7 (64/362)	0.57 (0.32–0.99)	0.0324	11.0 (24/218)	16.9 (66/402)	0.61 (0.35–1.06)	0.0612
Overweight (BMI/95th percentile)	8.1 (10/123)	5.5 (11/201)	1.53 (0.63–3.71)	0.3661	4.8 (6/124)	8.3 (19/228)	0.56 (0.22–1.43)	0.1836
Low birth weight (< 2,500 g)	3.8 (8/211)	5.0 (18/357)	0.74 (0.31–1.78)	0.4804	2.8 (6/214)	5.3 (21/399)	0.52 (0.19–1.40)	0.1793
Premature birth	10.4 (22/212)	12.2 (44/362)	0.84 (0.44–1.57)	0.5749	8.7 (19/218)	11.9 (48/402)	0.70 (0.34–1.44)	0.3216
Early respiratory infection	13.7 (29/212)	9.9 (36/362)	1.44 (0.80–2.57)	0.2446	12.8 (28/218)	10.7 (43/402)	1.23 (0.68–2.23)	0.5049
NICU admission	9.0 (19/212)	12.2 (44/362)	0.71 (0.38–1.33)	0.2660	11.5 (25/218)	11.7 (47/402)	0.98 (0.54–1.76)	0.9418
High-risk birth*^a^*	17.0 (36/212)	22.4 (81/362)	0.71 (0.44–1.15)	0.1545	19.3 (42/218)	20.9 (84/402)	0.90 (0.56–1.45)	0.6730
Doctor-diagnosed asthma	13.2 (28/212)	10.5 (38/362)	1.30 (0.69–2.43)	0.4234	11.9 (26/218)	11.7 (47/402)	1.02 (0.55–1.91)	0.9433
Asthma/medications for wheezing	17.0 (36/212)	15.2 (55/362)	1.14 (0.67–1.95)	0.6301	17.9 (39/218)	15.7 (63/402)	1.17 (0.71–1.95)	0.5427
Current wheeze	19.3 (41/212)	18.2 (66/362)	1.08 (0.65–1.77)	0.7769	19.3 (42/218)	20.2 (81/402)	0.95 (0.58–1.53)	0.8194
Cough with exercise	18.4 (39/212)	19.3 (70/362)	0.94 (0.58–1.53)	0.8022	19.7 (43/218)	18.9 (76/402)	1.05 (0.65–1.72)	0.8331
FVC*^b^*	3.38 ± 1.20	3.34 ± 1.11	1.96 (1.07–3.58)	0.03	3.47 ± 1.18	3.25 ± 1.09	1.64 (0.90–3.01)	0.11
FEV_1_*^b^*	2.88 ± 0.96	2.88 ± 0.97	1.30 (0.67–2.52)	0.44	2.98 ± 0.95	2.78 ± 0.94	1.54 (0.77–3.08)	0.22
FEV_1_/FVC*^b^*	86.20 ± 7.09	86.48 ± 7.11	0.97 (0.93–1.02)	0.26	86.47 ± 6.99	86.88 ± 6.24	1.02 (0.97–1.06)	0.52
FEF 25th–75th percentile*^b^*	3.20 ± 1.12	3.23 ± 1.12	0.91 (0.64–1.29)	0.60	3.32 ± 1.10	3.07 ± 1.20	1.19 (0.84–1.68)	0.33
Positive methacholine challenge	49.2 (64/130)	52.0 (120/231)	0.90 (0.57–1.40)	0.6308	49.6 (69/139)	53.9 (137/254)	0.84 (0.54–1.31)	0.4445

Abbreviations: CI, confidence interval; BMI, body mass index; FEF, forced expiratory flow; NICU, neonatal intensive care unit; OR, odds ratio.

a High-risk birth is defined as premature birth, hospitalization in an NICU, use of oxygen following birth (not including resuscitation at birth), or use of oxygen at home after leaving the hospital.

b Adjusted for age, height, and sex.

**Table 2:** Farm exposures and environmental risk factors for living on a farm [% (no./total)].

Variable	Born on a farm	Not born on a farm	OR (95% CI)	*p*-Value	Currently lives on a farm	Does not currently live on a farm	OR (95% CI)	*p*-Value
Born on a farm	—	—	—	—	80.3 (171/213)	12.1 (46/381)	29.65 (16.63–52.86)	< 0.0001
Lived on farm for at least 3 months before 1 year of age	98.1 (212/216)	4.0 (15/375)	1,272 (342.50–4724.07)	< 0.0001	82.1 (174/212)	14.0 (53/379)	28.16 (15.90–49.88)	< 0.0001
Lived on farm for at least 3 months before 5 years of age	99.1 (214/216)	11.2 (42/375)	848.36 (203.16–3542.64)	< 0.0001	87.7 (186/212)	18.5 (70/379)	31.58 (16.95–58.84)	< 0.0001
Farm residence	78.8 (171/217)	11.1 (42/377)	29.65 (16.63–52.86)	< 0.0001	—	—	—	—
Parent does farm work	79.3 (172/217)	27.8 (105/377)	9.90 (5.80–16.90)	< 0.0001	95.1 (213/224)	20.2 (85/420)	76.32 (27.42–212.41)	< 0.0001
Maternal smoking during pregnancy	21.2 (45/212)	29.0 (105/362)	0.66 (0.36–1.20)	0.1467	18.4 (40/218)	31.6 (127/402)	0.49 (0.25–0.93)	0.0161
Current household exposure to tobacco smoke	13.5 (28/208)	26.1 (94/360)	0.44 (0.23–0.83)	0.0057	10.8 (23/214)	30.8 (123/400)	0.27 (0.13–0.54)	0.0001
Ever household exposure to tobacco smoke	20.7 (43/208)	42.3 (153/362)	0.36 (0.21–0.62)	0.0001	13.1 (28/214)	47.5 (191/402)	0.17 (0.09–0.32)	< 0.0001
Gas stove in home for cooking	48.7 (95/195)	46.4 (161/347)	1.10 (0.66–1.84)	0.7232	46.8 (95/203)	46.6 (176/378)	1.01 (0.58–1.75)	0.9730
Burn wood for fuel	31.3 (61/195)	20.8 (72/347)	1.74 (0.97–3.11)	0.0728	32.0 (65/203)	20.9 (79/378)	1.78 (0.97–3.28)	0.0680
Current dehumidifier use in home	54.4 (106/195)	30.8 (107/347)	2.67 (1.59–4.49)	0.0003	55.2 (112/203)	29.6 (112/378)	2.92 (1.66–5.15)	0.0002
Parent education (highest years of school)*^a^*	14.2 ± 2.1 (*n* = 215)	13.5 ± 2.0 (*n* = 377)	1.17 (1.04–1.32)	0.01	14.3 ± 2.0 (*n* = 222)	13.5 ± 1.9 (*n* = 412)	1.22 (1.07–1.39)	< 0.01
Household income (< $20,000)	2.4 (5/204)	10.6 (38/360)	0.21 (0.04–1.16)	0.0068	2.8 (6/211)	11.3 (45/399)	0.23 (0.05–1.03)	0.0084
Household pets: cats	66.7 (130/195)	49.0 (170/347)	2.08 (1.25–3.48)	0.0045	66.5 (135/203)	49.2 (186/378)	2.05 (1.19–3.54)	0.0092
Household pets: dogs	69.2 (135/195)	64.8 (225/347)	1.22 (0.69–2.16)	0.4869	70.9 (144/203)	65.3 (247/378)	1.29 (0.71–2.37)	0.3898
Applied pesticides in home during past year	57.4 (112/195)	58.2 (202/347)	0.97 (0.58–1.62)	0.9035	58.6 (119/203)	58.7 (222/378)	1.00 (0.57–1.74)	0.9873
Applied pesticides outside home during past year	49.7 (97/195)	33.4 (116/347)	1.97 (1.17–3.33)	0.0130	49.8 (101/203)	33.6 (127/378)	1.96 (1.12–23.43)	0.0220
Raise swine	40.4 (76/188)	3.8 (14/366)	17.06 (7.55–38.58)	< 0.0001	52.5 (96/183)	0.0 (0/420)	NA	< 0.0001
Raise livestock	68.6 (129/188)	7.4 (27/366)	27.45 (14.66–51.40)	< 0.0001	89.6 (164/183)	0.0 (0/420)	NA	< 0.0001
Add antibiotics in feed	27.1 (51/188)	3.6 (13/366)	10.11 (4.24–24.08)	< 0.0001	37.7 (69/183)	0.0 (0/420)	NA	< 0.0001

Abbreviations: CI, confidence interval; NA, not applicable; OR, odds ratio.

a Mean ± SD (no./total).

**Table 3:** Outcomes and environmental risk factors [% (no./total) or mean ± SD] for doctor-diagnosed asthma and asthma medications for wheeze.

Variable	Doctor-diagnosed asthma (*n* = 72)	Nonasthmatic (*n* = 538)	OR (95% CI)	*p*-Value	Asthma/medications for wheeze (*n* = 101)	Nonasthmatic (*n* = 509)	OR (95% CI)	*p*-Value
Parent education (highest years of school)	13.2 ± 1.9 (*n* = 71)	13.9 ± 2.0 (*n* = 533)	0.90 (0.80–1.02)	0.08	13.5 ± 1.9 (*n* = 99)	13.9 ± 2.0 (*n* = 505)	0.86 (0.73–1.02)	0.10
Raise swine	23.6 (17/72)	15.0 (76/507)	1.75 (0.85–3.63)	0.1861	24.0 (24/100)	14.4 (69/479)	1.88 (1.02–3.45)	0.0762
Add antibiotics in feed	15.3 (11/72)	10.8 (55/507)	1.48 (0.68–3.24)	0.3707	19.0 (19/100)	9.8 (47/479)	2.16 (1.15–4.04)	0.0407

Abbreviations: CI, confidence interval; OR, odds ratio. No significant association (*p* < 0.1) was observed for any asthma outcome for the following variables: farm residence, born on a farm, lived on a farm for at least 3 months while < 1 year of age, lived on farm for at least 3 months while < 5 years of age, parent does farm work, maternal smoking during pregnancy, current household exposure to tobacco smoke, ever household exposure to tobacco smoke, gas stove in home for cooking, burn wood for fuel, current dehumidifier use in home, household income (< $20,000), household pets: cats, household pets: dogs, applied pesticides in home during past year, applied pesticides outside home during past year, or raise livestock.

**Table 4:** Outcomes and environmental risk factors [% (no./total)] for current wheeze and cough with exercise.

Variable	Current wheeze (*n* = 120)	None (*n* = 490)	OR (95% CI)	*p*-Value	Cough with exercise (*n* = 117)	None (*n* = 493)	OR (95% CI)	*p*-Value
Burn wood for fuel	21.6 (24/111)	25.8 (117/454)	0.79 (0.46–1.37)	0.3896	16.8 (18/107)	26.9 (123/458)	0.55 (0.31–0.97)	0.0255
Household pets: dogs	67.6 (75/111)	67.6 (307/454)	1.00 (0.62–1.62)	0.9921	76.6 (82/107)	65.5 (300/458)	1.73 (1.01–2.94)	0.0350
Applied pesticides outside home during past year	33.3 (37/111)	41.8 (190/454)	0.69 (0.43–1.11)	0.1255	29.9 (32/107)	42.6 (195/458)	0.58 (0.35–0.96)	0.0282
Raise swine	20.3 (24/118)	15.0 (69/461)	1.45 (0.79–2.65)	0.2636	22.8 (26/114)	14.4 (67/465)	1.76 (0.97–3.19)	0.0970
Add antibiotics in feed	14.4 (17/118)	10.6 (49/461)	1.42 (0.74–2.71)	0.3328	17.5 (20/114)	9.9 (46/465)	1.94 (1.00–3.77)	0.0917

Abbreviations: CI, confidence interval; OR, odds ratio. No significant association (*p* < 0.1) was observed for any asthma outcome for the following variables: farm residence, born on a farm, lived on farm for at least 3 months while < 1 year of age, lived on farm for at least 3 months while < 5 years of age, parent does farm work, maternal smoking during pregnancy, current household exposure to tobacco smoke, ever household exposure to tobacco smoke, gas stove in home for cooking, current dehumidifier use in home, parent education (highest years of school), household income (< $20,000), household pets: cats, applied pesticides in home during past year, and raise livestock.

**Table 5:** Doctor-diagnosed asthma and asthma/medication for wheeze, family and personal risk factors, and respiratory symptoms and function [% (no./total) or mean ± SD].

Variable	Doctor-diagnosed asthmatic (*n* = 73)	Nonasthmatic (*n* = 538)	OR (95% CI)	*p*-Value	Asthma/medication for wheeze (*n* = 101)	Nonasthmatic (*n* = 509)	OR (95% CI)	*p*-Value
Male sex	72.6 (53/73)	51.6 (282/547)	2.49 (1.31–4.72)	0.0021	71.6 (73/102)	50.6 (262/518)	2.46 (1.46–4.13)	0.0003
Age (years)	11.0 ± 4.4	9.3 ± 4.9	1.1 (1.03–1.13)	< 0.01	9.5 ± 4.8	9.5 ± 4.9	1.0 (0.96–1.04)	0.96
No. of siblings < 18 years of age	1.5 ± 1.0	1.5 ± 1.1	0.97 (0.75–1.26)	0.81	1.4 ± 1.0	1.5 ± 1.1	0.93 (0.74–1.16)	0.52
Atopy (IgE)	56.7 (17/30)	32.6 (58/178)	2.71 (1.22–6.00)	0.0235	54.3 (19/35)	32.4 (56/173)	2.86 (1.35–6.05)	0.0208
Atopy (SPT)	30.8 (12/39)	16.2 (43/266)	2.30 (1.03–5.18)	0.0824	34.1 (15/44)	15.3 (40/261)	1.61 (0.83–3.15)	0.1671
SPT (mean positive)	1.46	0.98		0.0493	1.45	0.67		0.0286
Atopy (by questionnaire)	43.8 (32/73)	21.6% (118/547)	2.84 (1.43–5.62)	0.0172	41.2 (42/102)	20.8 (108/518)	2.66 (1.49–4.74)	0.0063
Diagnosed allergies	39.7 (29/73)	11.5 (63/547)	5.06 (2.92–8.77)	< 0.0001	39.2 (40/102)	10.0 (52/518)	5.78 (3.46–9.66)	< 0.0001
Overweight (BMI > 95th percentile)	9.6 (5/52)	6.7 (19/285)	1.49 (0.54–4.14)	0.4927	8.8 (5/57)	6.8 (19/280)	1.32 (0.48–3.66)	0.6183
Low birth weight (< 2,500 g)	6.8 (5/73)	4.1 (22/540)	1.73 (0.60–5.02)	0.3798	4.9 (5/102)	4.3 (22/511)	1.15 (0.40–3.31)	0.8066
Premature birth	20.6 (15/73)	9.5 (52/547)	2.46 (1.21–5.00)	0.0513	21.6 (22/102)	8.7 (45/518)	2.89 (1.60–5.23)	0.0066
NICU admission	19.2 (14/73)	10.6 (58/547)	2.00 (0.98–4.10)	0.1128	18.6 (19/102)	10.2 (53/518)	2.01 (1.07–3.78)	0.0603
High-risk birth*^a^*	34.2 (25/73)	18.5 (101/547)	2.30 (1.33–3.97)	0.0145	35.3 (36/102)	17.4 (90/518)	2.59 (1.61–4.19)	0.0011
Early respiratory infection	21.9 (16/73)	10.0 (55/547)	2.51 (1.23–5.14)	0.0463	21.6 (22/102)	9.5 (49/518)	2.63 (1.42–4.88)	0.0124
FVC*^b^*	3.45 ± 1.18	3.32 ± 1.13	0.69 (0.27–1.77)	0.44	3.42 ± 1.17	3.31 ± 1.13	0.63 (0.25–1.58)	0.32
FEV_1_*^b^*	2.87 ± 1.00	2.84 ± 0.94	0.43 (0.15–1.27)	0.13	2.84 ± 0.97	2.85 ± 0.95	0.37 (0.13–1.06)	0.07
FEV_1_/FVC *^b^*	83.55 ± 7.29	86.40 ± 6.38	0.95 (0.90–1.01)	0.08	83.40 ± 7.57	86.48 ± 6.27	0.95 (0.90–1.00)	0.07
FEF 25th–75th percentile*^b^*	2.99 ± 1.21	3.18 ± 1.15	0.66 (0.39–1.10)	0.11	2.93 ± 1.18	3.19 ± 1.16	0.62 (0.37–1.02)	0.06
Positive methacholine challenge	63.6 (35/55)	51.4 (164/319)	1.65 (0.91–3.02)	0.0960	65.6 (40/61)	50.8 (159/313)	1.84 (1.03–3.30)	0.0343

Abbreviations: BMI, body mass index; CI, confidence interval; FEF, forced expiratory flow; NICU, neonatal intensive care unit; OR, odds ratio.

a High-risk birth is defined as premature birth, hospitalization in an NICU, use of oxygen following birth (not including resuscitation at birth), or use of oxygen at home after leaving the hospital.

b Adjusted for age, height, and sex.

**Table 6:** Current wheeze and chronic cough, family and personal risk factors, and respiratory symptoms and function [% (no./total) or mean ± SD].

Variable	Current wheeze (*n* = 120)	None (*n* = 490)	OR (95% CI)	*p*-Value	Cough with exercise (*n* = 117)	No cough (*n* = 493)	OR (95% CI)	*p*-Value
Male sex	56.9 (70/123)	53.3 (265/497)	1.16 (0.77–1.74)	0.4839	66.4 (79/119)	51.1 (256/501)	1.89 (1.22–2.93)	0.0046
Age (years)	8.0 ± 4.9	9.9 ± 4.8	0.93 (0.89–0.97)	< 0.01	10.7 ± 4.5	9.2 ± 5.0	1.06 (1.02–1.11)	< 0.01
No. of siblings < 18 years of age	1.4 ± 1.0	1.5 ± 1.1	0.89 (0.72–1.11)	0.30	1.4 ± 0.9	1.6 ± 1.1	0.85 (0.69–1.05)	0.13
Atopy (IgE)	45.4 (15/33)	34.3 (60/177)	1.60 (0.80–3.19)	0.2030	35.3 (18/51)	36.3 (57/157)	0.96 (0.48–1.91)	0.9000
Atopy (SPT)	45.4 (20/44)	13.4 (35/261)	5.38 (2.68–10.79)	0.0004	29.7 (19/64)	14.9 (36/241)	2.40 (1.29–4.49)	0.0145
SPT (mean positive)	1.95	0.58		0.0005	1.38	0.62		0.0097
Atopy (by questionnaire)	26.0 (32/123)	23.7 (118/497)	1.13 (0.66–1.95)	0.6676	26.0 (31/119)	23.8 (119/501)	1.13 (0.66–1.94)	0.6593
Diagnosed allergies	30.9 (38/123)	10.9 (54/497)	3.67 (2.25–5.97)	< 0.0001	30.2 (36/119)	11.2 (56/501)	3.45 (2.16–5.49)	< 0.0001
Overweight (BMI > 95th percentile)	13.0 (7/54)	6.0 (17/283)	2.33 (0.92–5.92)	0.1509	12.0 (9/75)	5.7 (15/262)	2.25 (0.96–5.25)	0.1143
Low birth weight (< 2,500 g)	6.5 (8/123)	3.9 (19/490)	1.72 (0.75–3.95)	0.2752	6.0 (7/117)	4.0 (20/496)	1.51 (0.60–3.85)	0.4182
Premature birth	17.1 (21/123)	9.3 (46/497)	2.02 (1.14–3.59)	0.0399	18.5 (22/119)	8.9 (45/501)	2.30 (1.23–4.31)	0.0243
NICU admission	15.4 (19/123)	10.7 (53/497)	1.53 (0.85–2.76)	0.1892	18.5 (22/119)	9.9 (50/501)	2.05 (1.14–3.67)	0.0395
High-risk birth*^a^*	27.6 (34/123)	18.5 (92/497)	1.68 (1.05–2.68)	0.0413	31.9 (38/119)	17.6 (88/501)	2.20 (1.38–3.51)	0.0033
Early respiratory infection	17.9 (22/123)	9.9 (49/497)	1.99 (1.10–3.60)	0.0487	18.5 (22/119)	9.8 (49/501)	2.09 (1.20–3.64)	0.0232
FVC*^b^*	3.35 ± 1.10	3.33 ± 1.14	0.94 (0.41–2.15)	0.88	3.47 ± 1.08	3.29 ± 1.15	0.90 (0.46–1.73)	0.74
FEV_1_*^b^*	2.81 ± 0.89	2.85 ± 0.96	0.60 (0.27–1.33)	0.21	2.90 ± 0.89	2.83 ± 0.97	0.57 (0.29–1.14)	0.11
FEV_1_/FVC*^b^*	84.27 ± 6.87	86.26 ± 6.52	0.95 (0.91–1.00)	0.06	83.94 ± 7.02	86.51 ± 6.38	0.95 (0.91–0.99)	0.01
FEF 25th–75th percentile*^b^*	2.98 ± 1.13	3.18 ± 1.17	0.69 (0.47–1.02)	0.06	3.05 ± 1.16	3.17 ± 1.17	0.69 (0.49–0.99)	0.04
Positive methacholine challenge	60.7 (34/56)	51.9 (165/318)	1.43 (0.81–2.54)	0.2160	61.0 (50/82)	51.0 (149/292)	1.50 (0.89–2.52)	0.1214

Abbreviations: BMI, body mass index; CI, confidence interval; FEF, forced expiratory flow; NICU, neonatal intensive care unit; OR, odds ratio.

a High-risk birth is defined as premature birth, hospitalization in an NICU, use of oxygen following birth (not including resuscitation at birth), or use of oxygen at home after leaving the hospital.

b Adjusted for age, height, and sex.

**Table 7:** Multivariable models for asthma outcomes.

	Doctor-diagnosed asthma		Asthma/medication for wheeze		Current wheeze		Cough with exercise	
Parameter	OR (95% CI)	*p*-Value	OR (95% CI)	*p*-Value	OR (95% CI)	*p*-Value	OR (95% CI)	*p*-Value
Male sex	2.62 (1.38–4.95)	< 0.01	2.41 (1.38–4.22)	< 0.01	—	—	1.75 (1.07–2.86)	0.03
Child’s age	1.09 (1.03–1.15)	0.01	—	—	0.93 (0.88–.097)	< 0.01	1.07 (1.02–1.13)	0.01
Ever been diagnosed with allergies	4.60 (2.56–8.25)	< 0.01	5.48 (3.10–9.70)	< 0.01	3.88 (2.26–6.66)	< 0.01	3.34 (1.97–5.67)	< 0.01
Atopy (by questionnaire)	2.58 (1.22–5.45)	0.01	2.40 (1.24–4.65)	0.01	—	—	—	—
Premature birth	2.43 (1.16–5.12)	0.02	—	—	—	—	—	—
Early respiratory infection	—	—	1.92 (0.87–4.23)	0.10	1.84 (0.92–3.70)	0.09	1.91 (1.01–3.62)	0.05
High-risk birth	—	—	2.08 (1.23–3.52)	0.01	—	—	2.13 (1.30–3.48)	< 0.01
Add antibiotics to feed and raise swine	—	—	2.47 (1.29–4.74)	0.01	1.91 (0.98–3.73)	0.06	2.72 (1.34–5.52)	0.01
Household pets: dogs	—	—	—	—	—	—	1.73 (0.98–3.06)	0.06

Abbreviations: —, risk factors not selected in the stepwise logistic regression; OR, odds ratio.
